# Vaccination of White-Tailed Deer with *Mycobacterium bovis* Bacillus Calmette–Guérin (BCG): Effect of *Mycobacterium avium* ssp. *paratuberculosis* Infection

**DOI:** 10.3390/microorganisms11102488

**Published:** 2023-10-04

**Authors:** Mitchell V. Palmer, Carly Kanipe, Kimberly A. Lehman, Tyler C. Thacker, Ellie J. Putz, Paola M. Boggiatto

**Affiliations:** 1 Bacterial Diseases of Livestock Research Unit, National Animal Disease Center, Agricultural Research Service, USDA, 1920 Dayton Avenue, Ames, IA 50010, USA; carly.kanipe@usda.gov (C.K.); ellie.putz@usda.gov (E.J.P.); paola.boggiatto@usda.gov (P.M.B.); 2 Immunobiology Graduate Program, College of Veterinary Medicine, Iowa State University, 1800 Christensen Drive, Ames, IA 50010, USA; 3 National Veterinary Services Laboratories, Animal and Plant Health Inspection Service, USDA, 1920 Dayton Avenue, Ames, IA 50010, USA; kimberly.lehman@usda.gov (K.A.L.); tyler.thacker@usda.gov (T.C.T.)

**Keywords:** BCG, Johne’s disease, mycobacteria, paratuberculosis, tuberculosis, white-tailed deer

## Abstract

In many parts of the world, bovine tuberculosis eradication efforts are hampered by wildlife reservoirs of *Mycobacterium bovis*, which serve as a constant source of *M. bovis* for nearby cattle. The human tuberculosis vaccine, *M. bovis* BCG has been investigated for use in several wildlife species, including deer. In the US, white-tailed deer in Michigan have been the source of infection for over 82 cattle herds since *M. bovis* was discovered in free-ranging deer in 1995. The efficacy of BCG may be influenced by many factors, including prior exposure or infection with non-tuberculous mycobacteria, that is, species other than members of the *M. tuberculosis* complex. *M. avium* subspecies *paratuberculosis* (*Map*) infection is not uncommon in ruminants such as deer. Using natural exposure to *Map* and experimental infection with *M. bovis,* we demonstrate that *Map* infection increased BCG vaccine efficacy as measured by lesion severity scores.

## 1. Introduction

Bacteria of the genus *Mycobacterium* are Gram-positive, acid-fast bacilli (AFB) comprising over 120 species, which are diverse in terms of host adaptation, pathogenicity, and growth characteristics [1]. The *M. tuberculosis* complex (MTBC) includes *M. tuberculosis*, *M. bovis*, *M. africanum*, *M. microti*, *M. caprae*, *M. canetii*, *M. pinnipedii*, *M. orygis*, *M. suricattae*, *M. mungi*, the dassie bacillus, and the chimpanzee bacillus [2,3,4,5,6,7,8]. Species other than MTBC, *M. leprae* and *M. lepromatous* [9], are known as non-tuberculous mycobacteria (NTM) [1,10,11,12].

Of the *M. tuberculosis* complex, *M. bovis* has the broadest host range, encompassing most mammals, including humans and cattle. Most developed countries have conducted long, costly campaigns to eradicate bovine tuberculosis (bTB) with varying degrees of success [13]. In cases where a wildlife reservoir of *M. bovis* infection exists, eradication has been impeded by the persistent transmission of *M. bovis* from livestock to wildlife (spillover) and subsequent transmission from wildlife back to livestock (spillback) [14]. Indeed, no country with an established wildlife reservoir of *M. bovis* has been successful in eradicating bTB from its cattle population. In northeast Michigan, USA, white-tailed deer (*Odocoileus virginianus*) serve as a recognized wildlife reservoir of *M. bovis* and a constant source of infection for cattle. First recognized in 1995, deer have been linked to infections in over 82 cattle herds, with 2–3 additional herds identified each year. Initially, control efforts, including increased deer harvest through hunting and the banning of supplemental feeding of deer, were effective in decreasing disease prevalence from 4.9% in 1995 to approximately 2%; however, the prevalence continues to remain near 2% [15,16,17].

A valuable tool to decrease deer-to-deer and deer-to-cattle transmission of *M. bovis* would be a vaccine. The most studied tuberculosis vaccine in deer, as well as other wildlife, is the attenuated strain of *M. bovis* known as bacillus Calmette–Guérin (BCG), named for Albert Calmette and Camille Guérin, two French scientists at the Pasteur Institute that developed the strain [18]. European badgers (*Meles meles*), brushtail possums (*Trichosurus vulpecula*), wild boar (*Sus scrofa*), African buffalo (*Syncerus caffer*), red deer (*Cervus elaphus*), and white-tailed deer are accepted wildlife maintenance hosts and reservoirs of *M. bovis*. In all these species, BCG vaccination does not generate sterile immunity, but rather results in decreased disease severity, as measured by fewer highly necrotic advanced granulomas, fewer gross lesions overall, as well as fewer tissues from which virulent *M. bovis* may be isolated. Thus, BCG vaccination may not prevent infection, but limited disease severity is hypothesized to decrease disease transmission [19,20,21,22,23]. 

The efficacy of BCG may be influenced by many factors, including host genetics, exposure dose, exposure route, vaccine strain, age at vaccination, concurrent disease, or exposure to NTM [24,25,26]. The reports on the effects of NTM exposure on BCG efficacy are inconclusive. Some studies suggest that preexisting sensitivities to NTM, including *M. avium*, either have no effect, confer some degree of protection, or decrease protection [24,27,28,29,30,31]. At one end of the spectrum, preexisting NTM exposure is believed to serve as a priming exposure resulting in a boosting effect when the BCG vaccine is administered; at the other end, NTM sensitivity interferes with BCG efficacy [24,31]. One proposed mechanism for reduced efficacy is that pre-existing immune sensitivity to NTM restricts BCG replication following vaccination, resulting in the dampening of critical cytokine responses such as that of interferon gamma (IFN-γ) [31].

In ruminants, the most commonly isolated NTM are members of the *M. avium* complex [32] which traditionally includes two closely related species, *M. avium* and *M. intracellulare*. There are four subspecies of *M. avium*: *hominissuis*, *avium*, *silvaticum,* and *paratuberculosis*. *Mycobacterium avium* sOsp. *paratuberculosis* (*Map*) is the cause of paratuberculosis, a chronic enteritis in domestic and wild ruminants, including cattle, goats, sheep, and deer, both captive and free-ranging [33,34,35,36,37,38,39,40,41,42,43,44,45,46].

Oral administration of BCG to white-tailed deer has been shown to reduce lesion severity scores compared to non-vaccinated deer [19,20,21,22]. As no biomarker of BCG vaccine efficacy exists in white-tailed deer, the evaluation of disease severity using lung and lymph node lesion scores is often used as a measure of vaccine efficacy. The objective of the present study was to evaluate the effect of prior natural *Map* exposure on lesion severity scores in deer orally vaccinated with BCG compared to those receiving no vaccination.

## 2. Materials and Methods

### 2.1. Deer, Vaccination, and Challenge

Fifteen 2–3-year-old female and castrated male white-tailed deer, shown previously by semi-annual direct fecal PCR and bacteriological culture of feces to shed *Map* were divided into 2 groups, BCG-vaccinated (n = 8) and non-vaccinated (n = 7). Similarly, 14 age-matched female and castrated male white-tailed deer were divided into 2 groups, BCG-vaccinated (n = 9) and non-vaccinated (n = 5). From birth, these 14 deer were raised apart, physically separated on a different pasture with no fence-line contact with *Map*-shedding deer. Semi-annual direct fecal PCR assays and fecal cultures were negative for *Map*. The pasture holding *Map*-shedding deer contained other deer known to be shedding *Map*, while the pasture holding *Map*-naïve deer had not held deer previously shown to be shedding *Map*, as determined through semi-annual fecal PCR and bacteriological culture. It was believed that this group of deer, as well as the pasture they occupied, were free of *Map*. Deer were orally vaccinated with *M. bovis* BCG Danish 1331 as previously described [19] with the aid of a swine mouth speculum. A 1.0 mL preparation of 9.3 × 10^7^ colony-forming unit (CFU) BCG in phosphate-buffered saline (PBS) was deposited in the posterior oropharynx using a 3 mL syringe and a 10 French urinary catheter (Monoject, St. Louis, MO, USA).

On day 167 post-vaccination, all deer were infected with a virulent, low passage field strain of *M. bovis* isolated from deer in Michigan, USA. Deer were anesthetized by the intramuscular (IM) injection of a combination of xylazine and ketamine. With the aid of a laryngoscope, 292 CFUs of *M. bovis* were deposited in both palatine tonsillar crypts for a total dose of 584 CFUs per deer. The effects of xylazine were then reversed by IM injection of tolazoline. Deer were housed inside high-containment biosecurity level 3 (BSL3) animal housing.

### 2.2. Postmortem Examination and Sample Collection

Deer were euthanized 152 days after infection and examined. The tissues collected were processed for both microscopic analysis and isolation of *Map* and *M. bovis*. These tissues included palatine tonsil, mandibular, parotid, medial retropharyngeal, tracheobronchial, and mediastinal lymph nodes, lung, distal jejunum, ileum, ileocecal valve, and lymph nodes associated with the ileocecal valve. Feces were also collected for direct PCR and *Map* isolation. Lymph nodes were sectioned at 0.5 cm intervals and examined. The lymph nodes most commonly containing tuberculous lesions in *M. bovis*-infected white-tailed deer are the medial retropharyngeal, tracheobronchial, and mediastinal nodes [47,48]. The scoring of gross lesions seen in these 3 nodes was performed as follows: (0) no visible lesions; (1) small focus (1–2 mm); (2) several small foci; and (3) extensive necrosis [49]. Each lung lobe was sectioned at 1.0 cm intervals and examined. Gross lung lesions were assessed based on the following scoring system: (0) no visible lesions; (1) no external lesions, but lesions seen on slicing; (2) <5 gross lesions of <10 mm; (3) >5 lesions of <10 mm; (4) >1 lesion of >10 mm; and (5) coalescing lesions [49]. Total lesion scores were obtained by adding the lesion scores of all 5 lung lobes and all 3 lymph nodes for each animal.

Tissue samples (≤0.5 cm in width) were fixed by immersion in 10% neutral-buffered formalin for 24 h, and then transferred to 70% alcohol followed by standard paraffin-embedding techniques. Paraffin-embedded samples were cut into 4-μm thick sections, transferred to Superfrost Plus charged microscope slides (Thermo Fisher, Waltham, MA, USA), and stained with hematoxylin and eosin (HE). Adjacent sections were stained by the Ziehl–Neelsen (ZN) technique for the visualization of AFB. Microscopic analysis of all tissues collected was performed. For each slide, all granulomas were staged (stages I–IV) according to criteria adapted from that described previously [50,51,52]. These stages are categorized as follows: initial (stage I), solid (stage II), necrotic (stage III), and necrotic and mineralized (stage IV).

All experimental animal procedures were conducted in accordance with the recommendations in the Care and Use of Laboratory Animals of the National Institutes of Health and the Guide for the Care and Use of Agricultural Animals in Research and Teaching [53,54] and approved by the institutional animal care and use committee.

### 2.3. M. bovis BCG Vaccine and M. bovis Challenge Inoculum

Virulent *M. bovis* and the BCG vaccine were grown separately in Middlebrook’s 7H9 media supplemented with 0.05% Tween 80 (Sigma Chemical Co., St. Louis, MO, USA) and 10% oleic acid–albumin–dextrose complex (OADC; Difco, Detroit, MI, USA) as previously described [55,56]. At the mid-log-phase growth stage, bacilli were pelleted by centrifugation at 750× *g*, washed twice with PBS 0.01 M, (pH 7.2), and stored at −80 °C. Frozen stocks were warmed to room temperature and diluted to the appropriate cell density in PBS. Bacilli were enumerated by serial dilution plate counting on Middlebrook’s 7H11 selective media (Becton Dickinson, Franklin Lakes, NJ, USA).

### 2.4. Culture of Feces and Tissues for the Isolation of Map

Tissues were stored frozen at −80 °C and processed as described previously for the isolation of *Map* [56]. For isolation of *Map* from feces, 2.0 ± 0.2 g of feces were added to 35 mL of sterile water in a 50 mL conical tube. Large clumps were broken up by vigorous shaking for at least 30 s, after which the sample was allowed to sit at room temperature (RT) for at least 30 min to allow debris to settle to the bottom of the tube. Five milliliters of the supernatant was transferred to a new 50 mL conical tube containing 25 mL of 0.9% hexadecylpyridinium chloride (HPC) solution and mixed thoroughly by vortexing. After overnight incubation at 37 °C, bacteria were collected by centrifugation at 900× *g* for 30 min. The supernatant was then discarded, and the pellet was resuspended in 1 mL of Johne’s antibiotic mix (100 µg/mL nalidixic acid, 100 µg/mL vancomycin, and 50 µg/mL amphotericin B in sterile water) and mixed thoroughly by vortexing for at least 15 s. The sample was then incubated overnight (12–24 h) at 37 °C. The pellet was resuspended by vortexing for a minimum of 15 s and subsequently inoculated into an in-house broth (Trek *para*-JEM broth; Thermo Fisher) supplemented with 1 mL of Trek *para*-JEM GS, 0.5 mL of Trek *para*-JEM AS, 0.05 mL of Trek *para*-JEM Blue, and 2 mL of 100% egg yolk. Samples were thoroughly mixed and incubated for up to 60 d (Versa TREK system; Thermo Fisher). Cultures were confirmed positive for *Map* by both ZN acid-fast staining and IS900 PCR, as described below.

### 2.5. Culture Confirmation Using PCR

To 500 mL of liquid culture was added proteinase K (100 µL, 10 mg/mL; MilliporeSigma, St. Louis, MO, USA), and the sample was incubated overnight at 50 °C. Bacteria were collected by centrifugation at 15,000× *g* for 15 min. Using a sterile cotton swab, the lipid layer was removed, and the remainder of the supernatant was removed using a pipette. The pellet was resuspended in 175 µL of phosphate-buffered saline (PBS). The resuspended pellet was then transferred to an O-ring screw-cap tube containing 400 µL of TE (Tris–EDTA; MilliporeSigma), 400 µL of phenol–chloroform–isoamyl alcohol (MilliporeSigma), ~125 µL of 1.0 mm glass beads (Biospec Products, Bartlesville, OK, USA), and ~1258 µL of 0.1 mm glass beads (Biospec Products). Disruption of bacilli was accomplished by shaking for 2 min in a bead beater (Biospec Products). The organic and aqueous phases were separated by centrifugation at 13,000× *g* for 10 min. Up to 400 µL of the aqueous phase (containing the DNA) was transferred to a fresh tube containing 1.2 mL of DNA-binding buffer (D6010-1-150; Zymo Research, Irvine, CA, USA) and mixed thoroughly. The DNA was captured using a spin column (Zymo-Spin IIC column C1011-50; Zymo Research) and centrifugation at 13,000× *g* for 1 min. The column was washed (200 µL DNA pre-wash buffer, D3004-5-50; Zymo Research) by centrifugation at 13,000× *g* for 1 min followed by a second wash (500 µL fecal DNA wash buffer, D6010-2-100; Zymo Research). The filter was transferred to a new tube, the DNA eluted (100 µL elution buffer, D3004-4-10; Zymo Research), and centrifuged at 8000× *g* for 1 min. The IS900 element was detected using the following PCR primers: 5′-AATCAACTCCAGCAGCGCGGCCTC and 5′-CCGCTAATTGAGAGATGCTGTAGG-3′ and probe 5′-/56-FAM/TCCACGCCC/ZEN/GCCCAGACAGG/31ABkFQ/-3′ in a qRT-PCR assay (PrimeTime Std; Integrated Technologies, Skokie, IL, USA) using TaqMan universal PCR master mix (Thermo Fisher) and 5 µL of isolated DNA according to the manufacturer’s instructions.

### 2.6. Direct Fecal PCR Testing

Fecal samples were maintained frozen at −80 °C until processed. A 300-μg sample of feces was placed into 1 mL of PBS and mixed thoroughly by vortexing for ~3 min. Solids were removed by centrifugation for 30 s at 100× *g*. Avoiding solid material, 175 mL of the supernatant was transferred to a tube containing 232 µL of MagMax lysis/binding solution (Thermo Fisher) prepared in accordance with the manufacturer’s instructions. These tubes also contained ~125 µL of 1.0 mm glass beads (Biospec Products), and ~1258 µL of 0.1 mm glass beads (Biospec Products). Bacilli were disrupted by shaking in a bead beater (Biospec Products) for 5 min. The sample was clarified by centrifugation at 16,000× *g* for 3 min. Using 20 µL of the clarified preparation DNA was isolated using the MagMAX total nucleic acid isolation kit; (Thermo Fisher), as instructed by the manufacturer. The IS900 element was detected by PCR, which was performed as described above.

### 2.7. Culture of Tissues for M. bovis

The processing of tissues for isolation of mycobacteria was performed as previously described with some modifications [57]. Tissues were trimmed, homogenized in saline, and decontaminated with 4% NaOH for 10 min. Once neutralized with a commercial buffer (IMMY, Norman, OK, USA), samples were centrifuged at 4600× *g*. The pellet was then inoculated into both BACTEC MGIT media and 7H11 Middlebrook with 0.5% hemolyzed blood, 10% calf serum, 0.39% sodium pyruvate, and 0.025% malachite green as additives. According to the manufacturer’s instructions, MGIT media were incubated appropriately. Signal-positive tubes were examined for the presence of AFB, which was confirmed by PCR. All PCR-positive MGIT samples were subcultured onto solid media, and suspicious colonies were identified as *M. bovis* by PCR, as described [58]. If MGIT media signaled positive prior to 42 days and no AFB were detected, incubation at 37 °C continued until day 42, at which time the sample was screened by PCR as described [59].

### 2.8. Statistics

Lesion scores between vaccinated and non-vaccinated deer were evaluated using the unpaired non-parametric Mann–Whitney test. When *Map* infection status and BCG vaccination status were both considered, the nonparametric Kruskal–Wallis ANOVA with Dunn’s multiple comparisons was used. Data are reported as median with interquartile range (GraphPad Prism 8.0, GraphPad Software, San Diego, CA, USA). For all analyses, a *p*-value < 0.05 was considered significant.

## 3. Results

### 3.1. Bacteriologic Culture

*Mycobacterium avium* subsp. *paratuberculosis* was isolated from the tissues of 15/15 (100%) *Map*-exposed deer. Unexpectedly, *Map* was isolated from the tissues of 6/14 (43%) assumed *Map*-naïve deer (Table 1). In all assumed *Map*-exposed deer, multiple tissues were positive for *Map*, the most common of which were the palatine tonsils (12/15), ileocecal lymph nodes (12/15), medial retropharyngeal lymph nodes (11/15), and ileocecal valve (8/15). By contrast, in the six assumed *Map*-naïve deer from which *Map* was isolated, with one exception, a single tissue was positive in each deer, the ileocecal lymph nodes being the most common (4/6), followed by the palatine tonsil (1/6).

Virulent *M. bovis* was isolated from at least one tissue from 17/29 deer; 10/17 (59%) vaccinated and 7/12 (58%) unvaccinated. The most common sites from which *M. bovis* was isolated were the palatine tonsils (8/17) and medial retropharyngeal lymph nodes (7/17), followed by lung (2/17) and parotid lymph nodes (1/17).

### 3.2. Gross Lesion Severity

**Figure 1:**
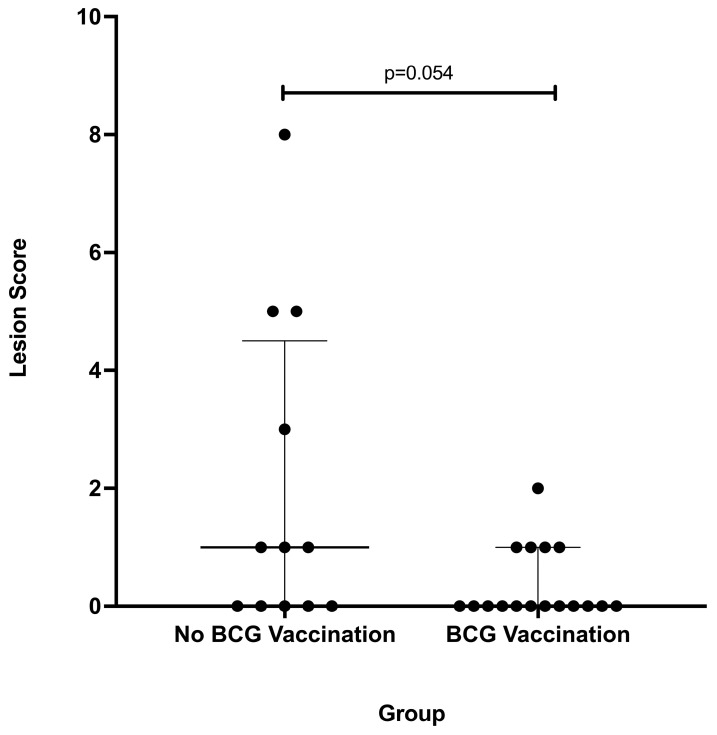
Total scores of lesions consistent with tuberculosis in white-tailed deer vaccinated orally with *M. bovis* BCG and experimentally infected with virulent *M. bovis*. Each dot represents an individual animal. Data are reported as the median with interquartile range of the sum of lymph node lesion and lung lobe lesion scores as outlined in the text. A *p*-value < 0.05 was considered significant.

Overall, 12/29 (41%) deer (5 vaccinates and 7 non-vaccinates) had gross lesions consistent with tuberculosis. The most common sites for gross lesions consistent with tuberculosis were the lung (7/12) and medial retropharyngeal lymph nodes (7/12), followed by the palatine tonsil (3/12). Although the number of deer with gross lesions was similar between vaccinates and non-vaccinates, differences in gross lesion severity scores in non-vaccinates compared to BCG vaccinates approached statistical significance (*p* = 0.054) (Figure 1).

Unexpectedly, the assumed *Map* status differed from the true *Map* infection status. When grouped by assumed *Map* infection status, based on antemortem fecal PCR and culture, total lesion scores were lower in BCG vaccinates shedding *Map*; however, this did not reach the level of significance (*p* = 0.262).

**Figure 2:**
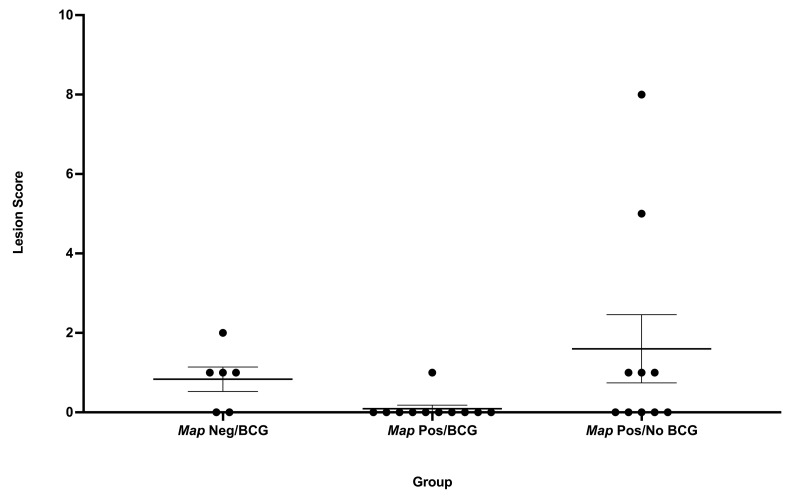
Total lesion scores based on true *Map* infection status in white-tailed deer vaccinated orally with *M. bovis* BCG and experimentally infected with virulent *M. bovis.* Each dot represents an individual animal. Data are reported as median with interquartile range of the sum of lymph node and lung lesion scores as outlined in the text. A *p*-value < 0.05 was considered significant.

When grouped by true *Map* infection status, as determined by the bacteriologic culture of numerous tissues, the non-parametric Kruskal–Wallis ANOVA showed *p* = 0.042. Although *Map*-positive deer that were vaccinated with BCG had lower lesion scores than deer vaccinated with BCG and from which *Map* could not be isolated (Figure 2), Dunn’s multiple comparisons test showed this difference was not significant (*p* = 0.084). Based on the true *Map* infection status, one group (*Map*-culture-negative, non-vaccinated deer) only contained two animals and was not considered for statistical analysis.

Microscopically, lesions consistent with *M. bovis* infection were characterized by variable infiltrates of epithelioid macrophages, lymphocytes, and multinucleated giant cells with variable amounts of central necrosis, mineralization, and peripheral fibrosis. When present, AFB were seen in very low numbers. Microscopic evaluation of tissue sections to determine the most severe stage of granuloma (I–IV) present within an animal revealed that only a single BCG-vaccinated deer had stage III–IV granulomas, while all but one of the non-vaccinates had stage III–IV granulomas present.

Microscopic lesions consistent with paratuberculosis (granulomatous infiltrates with intralesional AFB) were seen in samples of intestine and mesenteric lymph nodes examined in a single deer. In one *Map*-infected, BCG-vaccinated deer, infiltrates of macrophages containing numerous AFB expanded the villous lamina propria and submucosal regions of the distal jejunum, ileum, and ileocecal valve. Similar infiltrates were found multifocally in intestinal submucosal lymphoid structures, including Peyer’s patches and in lymph nodes associated with the ileocecal valve. Among these, lesions were most pronounced in the distal jejunum and ileum.

## 4. Discussion

Prior exposure to NTM has been suggested as a possible reason for the decreased efficacy of BCG vaccination in humans observed in certain geographic regions [60]. Previous studies of the effect of exposure/infection with NTM on BCG vaccine efficacy in animals have produced conflicting results. Some studies using mouse or guinea pig models of human tuberculosis suggest that preexisting sensitivities to NTM, including *M. avium*, have no effect or even confer some degree of protection [24,27,28,29,30]. Studies in cattle suggest that NTM sensitivity interferes with BCG vaccine efficacy [31]. Similar studies have not been conducted in deer, and the effects of NTM on BCG vaccination are unknown. Like other ruminants, deer are exposed to various NTM in their environment. In this regard, numerous NTM, including *Map* have been isolated from both free-ranging and captive deer species [20,21,22,61,62,63,64]. In the present study, although the true *Map* infection status was not what was assumed based on antemortem testing, it did allow us to compare the effect of *Map* infection on BCG vaccine efficacy. The data suggest that infection with *Map* may increase BCG vaccine efficacy rather than interfere with it, although this difference was not statistically significant. One of the limitations of this study is the small number of animals in each group, which likely impacted the ability to detect statistically significant differences. It would be beneficial to confirm the present findings using larger group sizes. This is especially critical given that the authors had to reclassify animals based on their true *Map* infection status after the experiment was concluded. Obtaining suitable numbers of white-tailed deer tame enough to be housed in BSL3 containment is challenging. Moreover, based on the results presented here, finding *Map*-free white-tailed deer may be even more difficult given the lack of sensitivity of current antemortem tests.

*Mycobacterium bovis* BCG might be used as a vaccine to control *M. bovis* infection in captive or free-ranging deer. The *Map* exposure/infection status of free-ranging deer is unclear and likely unknown. However, the presence of free-ranging deer on and around beef or dairy cattle operations is well known, and deer-to-cattle transmission of the related mycobacterial pathogen, *M. bovis*, is well documented [65,66,67]. Similarly, *Map*-infected deer have been identified on and around *Map*-infected beef and dairy cattle operations [37,46,68,69,70]. Moreover, a sampling of Minnesota dairy farms estimated that the probability of daily physical contact between cattle manure and deer was approximately 20% [70]. Paratuberculosis can also be a serious problem in farmed deer, especially where semi-intensive deer farming is practiced [34,71,72,73,74,75,76].

Good correlates of BCG-induced protection in ruminant studies do not exist. Measurements of immune response to vaccination such as intradermal tuberculin testing or cytokine production do not predict protection [23]. Postmortem evaluation of disease severity through lesion scoring, assessment of dissemination, histopathological staging, or quantitative bacteriological culture of key target tissues have all been used alone or in combination in cattle and deer BCG vaccine efficacy studies. The medial retropharyngeal lymph nodes have been the most common tissues from which *M. bovis* has been isolated in naturally infected deer [47,48]. Moreover, the palatine tonsils contain microscopic granulomas and harbor *M. bovis* in most naturally infected deer where lesions in the medial retropharyngeal lymph nodes are identified [77]. In the present study, similar to naturally infected deer, tuberculous lesions were most common in the medial retropharyngeal lymph nodes and palatine tonsils. Additionally, BCG vaccination resulted in lower total lesion scores (approaching statistical significance) in vaccinates compared to non-vaccinates, similar to previous studies [19,20,21,22]. Also, consistent with previous studies, late-stage III–IV granulomas were seen in all but one non-vaccinates and in only one vaccinate. Late-stage granulomas are highly necrotic and generally contain more AFB than early-stage granulomas and are believed to increase the risk of disease transmission. The paucity of late-stage granulomas in BCG vaccinates seen here supports some level of protection in BCG-vaccinated deer.

The finding of *Map* in the tissues of deer assumed to be *Map*-naïve was unexpected. Isolation of *Map* from the feces is considered the gold standard antemortem test for diagnosing *Map* infection in ruminants [78]. Even so, 2–3 years of semi-annual sampling of feces by direct PCR and bacteriological culture of presumed *Map*-naïve deer did not show evidence of *Map* infection. It is apparent that subclinical infections were present. In a previous report, *Map* was isolated from the tissues of white-tailed deer in a *Map*-endemic setting, even though several samples of feces had been PCR negative and culture negative for *Map* prior to necropsy [79]. Thus, it is apparent that *Map*-infected white-tailed deer may shed *Map* intermittently over long periods of time and that examination of feces by direct PCR and bacteriological culture, even when done semi-annually over a 2–3-year period, is not sufficient to identify all *Map*-infected deer. Other means of detection such as identification of *Map*-specific antibodies using ELISA or AGID assays have been used in white-tailed deer and other deer species to identify *Map*-infected animals with variable results, and are not considered reliable [45,78,80,81].

## 5. Conclusions

We conclude that, in the present study *Map* infection tended to enhance rather than interfere with BCG vaccine efficacy in white-tailed deer experimentally infected with *M. bovis*. However, the limitations of the present study include the limited number of animals in each treatment group, and reliance on lesion scoring and histopathological grading of granulomas as a measure of vaccine efficacy, which are admittedly relatively crude, but generally accepted measures of efficacy. Vaccination of captive or free-ranging deer with BCG may provide an additional tool to control tuberculosis.

## Figures and Tables

**Table 1:** Number of animals in each group based on assumed *Map* status (determined by semi-annual testing of feces by direct PCR and bacteriologic culture), compared to true *Map* infection status based on bacteriologic culture, followed by PCR confirmation, of numerous tissues at necropsy. n = number of animals.

Assumed Status	BCG/*Map* POS	No BCG/*Map* POS	BCG/*Map* NEG	No BCG/*Map* NEG	Total
n	8	7	9	5	29
True infection status	BCG/*Map* POS	No BCG/*Map* POS	BCG/*Map* NEG	No BCG/*Map* NEG	
n	11	10	6	2	29

## Data Availability

All data are available in the manuscript.
